# The effect of sex on suicide risk during and after psychiatric inpatient care in 12 countries—An ecological study

**DOI:** 10.1192/j.eurpsy.2020.83

**Published:** 2020-09-07

**Authors:** Stephan Listabarth, Benjamin Vyssoki, Alexander Glahn, Andrea Gmeiner, Nathalie Pruckner, Sandra Vyssoki, Andreas Wippel, Thomas Waldhoer, Daniel König

**Affiliations:** 1 Clinical Division of Social Psychiatry, Department of Psychiatry and Psychotherapy, Medical University of Vienna, Vienna, Austria; 2 Department for Psychiatry, Social Psychiatry and Psychotherapy, Medical University of Hannover, Hannover, Germany; 3 Department of Health Sciences, St. Pölten University of Applied Sciences, Sankt Pölten, Austria; 4 Center for Public Health, Department of Epidemiology, Medical University of Vienna, Vienna, Austria

**Keywords:** Gender, in-hospital suicide, postdischarge suicide, psychiatric care, suicide

## Abstract

**Background.:**

Suicide risk in patients is markedly elevated during psychiatric inpatient care, as well as after discharge. However, it is unclear whether, and to what extent, this increased suicide risk varies between sex. Thus, the aim of this study was to analyze sex differences for suicides during and after psychiatric hospitalization in various countries.

**Methods.:**

National suicide mortality rates and inpatient-related suicide rates (three intervals: during psychiatric inpatient treatment, 1 month, and 1 year after discharge) from 12 countries for 2000–2016 were analyzed, and a logistic model was used to quantify the effect of sex.

**Results.:**

Persons admitted to or discharged from psychiatric inpatient care exhibited significantly increased rates of suicide compared to those in the general population. Furthermore, increase of suicide risk was significantly higher for females than for males for all investigated time intervals (inpatient suicide odds ratio [OR] 1.85; suicide within 1 month after discharge—OR 1.94; suicide within 1 year after discharge—OR 2.04).

**Conclusion.:**

Analysis confirmed the time during and after psychiatric inpatient care to be significantly associated with an elevated risk for suicide. Further, a significant sex effect was observed, with females in this population being at a proportionally higher risk for suicide during psychiatric inpatient treatment as well as the year following discharge. Our study implicates that more effective suicide preventive measures during inpatient stay, focusing on female patients, are needed.

## Introduction

Suicide continues to be one of the most common causes of premature death and is responsible for close to 800,000 preventable deaths worldwide per year [1]. Suicidal behavior is the end point of a complex interplay of multiple risk factors [2]. As previously postulated, the time span during and after psychiatric inpatient treatment represents an important risk factor in this regard. Patients receiving inpatient psychiatric care are more severely affected by mental health disorders, when compared to the population for which outpatient care is sufficient and thus exhibit an increased risk for suicide [2–6]. In particular, the first week following discharge from inpatient psychiatric care was significantly associated with increase of suicidal behavior in previous studies [3,7–11]. Additionally, for up to 1 year after discharge, suicide was reported to be responsible for 59.6% of all former patients’ unnatural deaths [12]. Revealing relevant factors to identify persons more prone toward suicidal behavior is of major clinical relevance in order to establish more effective suicide preventive interventions in this high-risk population.

While there is consensus on profound sex differences in suicide rates in the general population, with higher rates of completed suicide for males being reported for most countries, there are limited data on the influence of sex on suicides during psychiatric inpatient care or after discharge from such [1,13]. Previous studies published contradictory findings, with some reporting females to be at increased risk for suicide after discharge [4,14,15], whereas others found males to be at an increased risk [3]. When investigating recurrent suicidal behavior, no significant sex difference was reported [16]. Further, previous studies were based on national or regional register data from single countries only.

This study aims to provide a more thorough analysis of sex differences in hospital-related suicide in patients in need of psychiatric care, by examining data from 12 countries for the time period of 17 years.

## Methods

### Data

Data for the time period from 2000–2016 were extracted for each country from the “Organisation for Economic Co-operation and Development’s Statistics Database (OECD Stats Database)” [17] and the “World Health Organisation — Health for All Database (WHO-HFA)” [18]. Suicide rates were obtained for (1) the general population, (2) population receiving inpatient psychiatric treatment, as well as (3) 1 month, and (4) 1 year after discharge from inpatient psychiatric care. Suicide rates were stated as number of suicides per 100,000 inhabitants per year for females and males separately, as well as combined. All countries for which the examined variables were available for this time period were included: Czech Republic, Denmark, Finland, Iceland, Israel, Latvia, Lithuania, Netherlands, Slovakia, Slovenia, Switzerland, and the United Kingdom.

### Statistical analysis

The aim of the statistical analysis was to compare overall national mortality rates due to suicide with standardized death rates (SDRs) due to suicide in patients currently receiving or having been discharged from inpatient psychiatric care for two separate time periods.

The following statistical methods were applied: Proportions of suicide per period were calculated by dividing the corresponding rate by the total suicide rate. Based on the national total number of suicides, the relative share of suicides of patients in need of psychiatric inpatient care was calculated per period (i.e., within inpatient stay, within 30 days, and within 1 year after discharge). The effect of time and sex was modeled by a logistic model including a fixed effect for sex and a random effect for country using the procedure “proc nlmixed” in SAS 9.4 (SAS Institute Inc., Cary, NC). The effect is described by the odds ratio (OR) and corresponding 95% confidence interval (CI). *p*-Values were adjusted for multiple tests by setting the significance level to 5/3 = 1.66% (*p* < 0.0166).

## Results

### Descriptive analysis

SDRs due to suicide differed widely between the 12 included countries, ranging from zero reported suicides per year for a specific time period up to a suicide rate of one suicide per 100 patients discharged found for the follow-up period of 1 year after discharge. Such particularly high numbers were observed in females and males in the Netherlands as well as for male population only in Slovenia. When analyzing median national death rates due to suicide across all countries, suicide rates were higher for males than for females across all observed time intervals (see Table 1).

**Table 1.** Pooled suicide rates (National SDR, National death rate due to suicide per 100,000 population; inpatient, suicide rate per 100,000 patients at inpatient care due to mental illness; 30 days after discharge, suicide rate per 100,000 patients within 30 days after discharge of inpatient care due to mental illness; 1 year after discharge, suicide rate per 100,000 patients within 1 year after discharge of inpatient care due to mental illness).

Sex	Suicide rate	Range	Mean	Median	Suicide rate/National SDR
All	National SDR	0–46.73	14.76	12.36	–
	Inpatient	0–310.00	68.32	70.00	5.67
	30 days after discharge	0–380.00	116.70	110.00	8.90
	1 year after discharge	0–1020.00	399.17	410.00	33.18
Females	National SDR	0–15.64	5.99	5.72	–
	Inpatient	0–330.00	50.28	40.00	6.99
	30 days after discharge	0–360.00	84.90	80.00	13.99
	1 year after discharge	0–1030.00	293.81	290.00	50.70
Males	National SDR	0–83.39	24.58	20.80	–
	Inpatient	0–390.00	90.19	80.00	3.85
	30 days after discharge	0–420.00	150.41	140.00	6.73
	1 year after discharge	0–1060.00	515.15	530.00	25.49

Abbreviation: SDR, standardized death rate.

### Statistical analysis—Suicide during and after psychiatric hospitalization

#### Model including country and sex

The statistical model including the variable sex, but not time, showed significant differences in likelihood of completed suicide for the population currently receiving inpatient psychiatric care as well as for those recently discharged from inpatient psychiatric care, when compared to the general population at adjusted levels of significance (*p* < 0.0166).

When the likelihood for completed suicide for patients currently receiving inpatient psychiatric care was analyzed, the proportion of females was found to exhibit a significant increase of likelihood for completed suicide (OR = 1.85; 95% CI [1.46, 2.34]), when compared to males.

**Figure 1.**
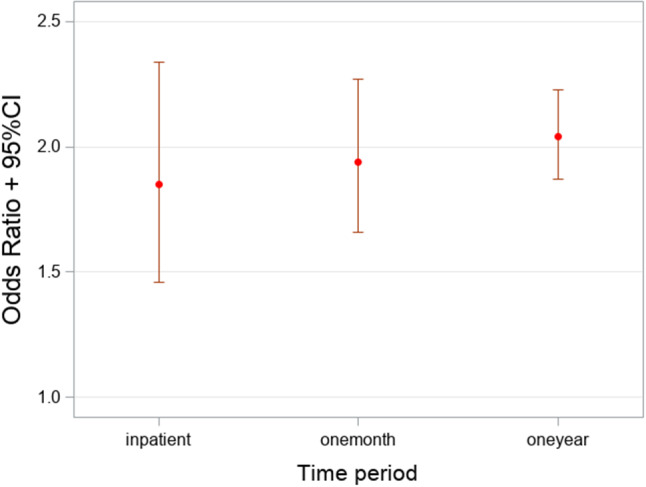
Odds ratios for suicide associated with treatment and discharge from hospitals due to mental illness and sex. ORs with values >1 represent a higher risk for suicide in females.

Furthermore, for the population of patients recently discharged from inpatient psychiatric care, a significantly increased proportion of likelihood of completed suicide in females compared to males was revealed for both intervals analyzed in our model—suicide within the first month after discharge (OR = 1.94; 95% CI [1.66, 2.27]) as well as suicide within the first year after discharge (OR = 2.04; 95% CI [1.87, 2.23]) (see Figure 1). Summarized, across all time intervals included in our analysis, the increase of proportion of suicides per period, compared to total suicide rate, was higher for females than for males.

A detailed description of suicide counts per period per country as well as frequencies is given in our Supplementary Table 1 in the provided supplementary material.

#### Model including country, sex, and time

The fixed effect of time turned out to be not significant at the adjusted level of significance (*p* < 0.0166) with corresponding *p*-values of 0.561, 0.023, and 0.133 for the three time periods.

## Discussion

In this study, we aimed to investigate the suicide rate during and after psychiatric inpatient treatment for 12 countries. In line with previous literature, the time period of psychiatric inpatient stay and the time period after discharge were significantly associated with an increased risk for suicide, with suicide rates up to 60 times higher than observed in the general population of the corresponding country [19].

In a next step, sex-specific suicide rates were then compared to those of the general population in order to investigate sex differences in the likelihood of completed suicide. These results revealed a marked gender gap: While in the general population, significantly higher rates of completed suicides in males are found, a finding which has also been extensively described in previous literature; our analysis showed that this sex effect is altered in the population currently receiving or being discharged from inpatient psychiatric care. Within these groups, the proportion of females among victims of completed suicide was significantly higher than in the general population across all 12 countries.

Conflicting data on the effect of sex on the rate of completed suicides, especially in the population of patients currently receiving inpatient psychiatric care, was previously published. In line with our findings, a large cohort study in Hong Kong identified females, recently discharged from inpatient psychiatric care, to be at increased risk for suicide within the first 28 days after discharge [14]. Further, a cohort study from Scotland reported suicides of female patients discharged from psychiatric hospitals to be responsible for an increased fraction of suicides in the total female population [4]. In contrast to our finding, a recent systematic meta-analysis of 13 case–control studies reported male sex to be associated with increased rates of postdischarge suicide [20]. However, some methodological differences exist compared to the current study, and while the aforementioned meta-analysis is comprised of studies from China, USA, Switzerland, and the United Kingdom, the present analysis focuses on European data, also incorporating data from Eastern European as well as Nordic countries and Israel. Besides the larger variation in included countries, the number of suicides included in the meta-analysis is rather low, with 1,554 total suicides.

These partially disagreeing findings in regard to sex differences in incidence of suicide are hypothesized to be caused by regional differences and sociocultural factors (e.g., economic turmoil, social structures/values, access to mental health care) exerting an important moderating effect [2,21]. Disagreeing with this hypothesis, however, no statistically significant differences for mortality rate due to suicide related to inpatient psychiatric treatment were found in our analysis, when data were analyzed for regional differences and for time trends over the past two decades.

Previous literature postulated additional hypotheses as to why female sex may be associated with an increased risk for suicide following inpatient psychiatric treatment: First, a gender gap in mental health care utilization, with females being more likely to seek psychiatric treatment and being hospitalized due to mental health conditions, is postulated [22–24]. This, in turn, may cause a selection bias, when considering males to be underrepresented within the population of inpatient psychiatric care, eventually resulting in increased relative suicide rates in females when compared to males.

Second, recent findings described females to be more likely than males to exhibit suicidal behavior, when exposed to stressful situations or life events. Thus, the increase of suicide rate in females could be hypothesized to be caused by this population being more severely affected by the stress of being admitted to/discharged from inpatient psychiatric care [25]. Furthermore, affective disorders, frequently associated with increased incidence of completed suicide, show higher rates of prevalence in females than in males, potentially contributing to an increased ratio of completed suicide in females currently receiving inpatient psychiatric care, when compared to the suicide rate of the total population [20,26,27].

Conversely, Möller-Leimkühler [13] proposed culturally defined gender models to impose increased psychosocial stress and maladaptive coping strategies on males within Western societies with males postulated to show higher rates of incidence for substance abuse and violent behavior following mental distress [28]. This hypothesized gender-related propensity for aggression is thought to explain higher rates of completed suicide for males in the general population [13,28].

Our findings, however, suggest that this hypothesis may not be applicable in the population of patients currently receiving inpatient psychiatric care or of those having recently been discharged from inpatient psychiatric care. Male individuals, admitted to psychiatric inpatient care, may receive more appropriate psychosocial support during psychiatric inpatient treatment, and this may, subsequently, increase the ratio of completed suicide risk of females. Interestingly, a recent study from Australia reported the rates of suicide for young females but not males to have significantly increased in recent years [29].

## Limitations of This Study

For this study, aggregated data from administrative registries were used, and thus, results should be interpreted with caution. Furthermore, data were only available for several selected countries. As a consequence, the results might not be applicable for countries not included. However, since the described effect of sex was very consistent and distinct within our data, it seems fair to assume that it applies to other countries, especially in the European region, as well.

## Conclusions

This study indicates sex as a highly relevant factor for suicidal behavior related to inpatient psychiatric care, with females being at a significantly increased risk when compared to males during inpatient treatment as well as for the following 12 months after discharge. This is in contrast to the otherwise higher risk for completed suicide in males in the general population, implying that females during and after inpatient psychiatric care are exposed to a deleterious factor, resulting in a significantly increased risk for completed suicide. Further research is needed to confirm these clinically relevant findings and also to provide potential explanations for this significant gender gap by examining causal pathways behind this phenomenon. In conclusion, these findings underline the importance of suicide preventative measures in the population of individuals treated in a psychiatric inpatient setting and show that more efficient measures are needed, especially for female patients.

## Data Availability

The dataset generated that support the findings are available from the corresponding author upon reasonable request.
